# Reports of Societies

**Published:** 1874-09

**Authors:** 


### Reports of Societies.

***Chicago Society of Physicians and Surgeons.*** *Transactions at Regular Meeting, Monday, July* 27, 1874. Reported by RALPH E. STARKWEATHER, M.D.

The Society met, as usual, in one of the parlors of the Grand Pacific Hotel, Dr. J. E. Owens, Vice-President, in the chair. After requesting Dr. H. A. Johnson to take the chair, Dr. Owens read a paper upon Thermometers, of which the following is a summary:

The experiments and observations had extended, at intervals, through three months, as many as fifty-five self-registering fever thermometers having been examined and tested. In view of the constant necessity for the use of this instrument at the bedside, the profession ought to know the character and worth of the fever thermometers offered for sale. Contrary to the popular supposition, these experiments have shown that the thermometer is not an unerring measure of temperature, unless proper care be exercised in selecting the thermometer. A large number, offered for sale in the shops, do not register correctly.

Every thermometer should be compared with a test instrument, one in all respects standard and reliable.

The indices having been shaken to the same level, approximately, each lot was tested in warm water. In one lot of instruments examined, it was found that no two registered alike; in another lot the errors were from three-fourths to one and one-half degrees Fah. Each instrument remained beneath the tongue four minutes in this trial. In a fourth collection, the index of one thermometer could not be moved, while that of another had been lost in the mercury below, and could not be disengaged; while no two gave the same registration, comparing them with a test thermometer made by Beck, of London, of ascertained reliability and accuracy. In a sixth lot of eleven, only one registered correctly.

The instruments above spoken of were mostly of American manufacture, not marked by their makers' names, and almost entirely unreliable. They are unworthy the patronage of the profession.

The English thermometers, made by Maw, Son, Thompson, and Lynch & Co., being tested, varied only by one-fifth of a degree, and are more worthy of confidence. All instruments that are not known to be correct should be tested with a Casella thermometer. Mr. E. H. Sargent, of this city, a prominent dealer in instruments, has allowed the use of the English thermometers, above spoken of.

Dr. Johnson—In regard to the index of a thermometer, I have long been satisfied that some thermometers will not always register the same in the same conditions of location and temperature. The mercury bubble adheres to the side of the tube and does not flow readily. Sometimes it goes up with a jump, and beyond its true level. There ought not to be a variation of even the one-half of a degree. Select an instrument in which the index will move readily; the more easily it does so, the more reliable it will be. If the index moves by starts, fitfully, it should be rejected.

Dr. Hyde, the Secretary, exhibited a cornu, removed from the forehead of an Irish washerwoman fifty-three years old, by Dr. McArthur. She had been operated upon by Dr. Andrews, three years before, who removed at that time a cystic tumor from the anterior part of the left frontal bone. It was presumed that part of the cyst had been left *in situ*—"the root," as she termed it—since the growth returned where the cicatrix had formed. Six months prior to date, her son had sawn off a portion of the horn as large as that which was removed by the knife. The patient presented herself at the Dispensary, not because she suffered any pain, but because the growth was a source of annoyance to her on account of the ridicule it excited among her neighbors. She has at present six or seven wens upon the occipital and parietal regions, which she refuses to have removed for fear that similar horns would subsequently appear in the cicatrices that would result. The specimen presented was nut-shaped at its extremity, and of even thickness.

As to the cause of the origin of the horns, Dr. Hyde said that Drs. Wilson and Fox regarded the cornu as a development of the sebaceous glands. Neuman, however, whose views of the pathology of this subject would be of great weight and authority, says that the growth is due to the development of the papilla of the corium. It resembles a wart or a corn. The sebaceous follicle is an appendage of the hair. The hair, nail, and tooth, are identical, so far as the type is concerned: all proceed from a papilla. A French writer has reported one case in which the cornu had three extremities.

Dr. W. C. Lyman stated that he had removed a cornu, an inch and one-quarter in length, from the centre of the lower lip.

Dr. Johnson exhibited a small electro-faradic machine, with a chloride of silver battery, combined in a pocket-case. It weighs only fourteen ounces. This battery does not need to be prepared each time it is required for use, there is no acid or fluid of any kind, and it is much cleaner than others. Its strength is sufficient to run the helix of the largest size of the Kidder apparatus, and may be moderated at pleasure. It will run continuously ten and even twelve hours before needing renewal. The induced current is sufficient for medical purposes. There is, also, the extra current; and either form may be used; or, thirdly, the current resultant of the first two may be used.

The battery itself is the chloride of silver and zinc. The elements are composed of a strip of zinc and another of chloride of silver. It is moistened by the chloride of sodium. These are enclosed in a hard rubber case, with a top screw on, by which it is hermetically sealed. Two elements go with each case, and can readily and quickly be detached from the rubber case when exhausted, and new ones substituted. The machine is set in motion by moving an armature. Metal handles, or electrodes, are also provided.

Dr. N. S. Davis then addressed the Society upon the subject of diarrhœa in children.

Dr. Hyde—In diarrhœa we all look at the stools, and compare them with our idea of a normal stool. Will Dr. Davis tell us what such ought to be, in a child eight to twelve months, in summer, and while teething?

Dr. Davis—My idea is that a normal stool would be the same as it would be in a cold day. Teething has no influence upon it. I cannot understand how it is that the teeth become poisonous in hot weather, when, in winter, dentition goes on harmlessly. A natural stool varies from a soft semi-fluid, up to a well-formed, consistent stool, retaining the shape of the intestine. If much beyond simple softness, it is abnormal. The color may be a little more yellow than usual. A great many slight cases of diarrhœa have stools of thinner consistence, unpleasant yellow color, and very offensive odor. As to the significance of the green stools, or their afterwards turning green, this may be due to the bile matter, but not always. The matter from the solitary glands of the large intestine may assume a green hue. You can test for bile in the stools.

Dr. Hyde had noticed that in classes of adults, those of different complexions had different color of stools. The same was true in regard to dogs; those white, had white-colored stools; those black, the Newfoundland, for instance, had black-colored stools. It is very much so in children. He laid less stress upon the color of stools than he did upon their homogeneity and consistence.

Dr. Davis—To prevent mistake, I will state that I look to the consistence and the homogeneity of the stools, and whether they are mucous. If the stools are consistent, I regard the patient as nearly well. The color is nothing, unless very markedly changed, when it will indicate suppression of the secretions, which must not be neglected. The more you physic a patient, the less likely you will be to have the true secretory organs collateral to the bowels acted upon, and the nearer you will approach to the true choleraic condition. Hence, if you want to act on the secretions collateral to the bowels, keep the latter quiet, and increase excretory action by minute doses of alteratives and diuretics. Many a child has been killed by trying to carry off the secretions by active cathartic medication.

Dr. F. H. Davis had seen the statement that the diarrhœa of children was unknown on the Continent, and was peculiar to American cities. Perhaps this suggests the origin of the disease. Some say it is malarial, and treat it with quinine, in combination with opiates.

Dr. Simon—It seems to me that there is some coincidence in the relation between the color of the fæces and the complexion. Physiology teaches that the pigment cells take their origin in the liver.

Dr. Oleson was referred to as treating infantile diarrhœa by the potassium bromide, believing that it operated by allaying the irritability of the mucous lining of the bowels.

Dr. N. S. Davis—Some three years ago some reliable writer proposed a similar treatment. I have used it in a few cases, alone, and, more often, combined with paregoric, in camphor water. The effects have been pleasant and favorable. In certain cases, where he could not gain time enough to control the bowels, and there were cerebral symptoms, he had used the bromide and paregoric. There need be no fear in using the bromide alone, but there would probably be need of the paregoric.

Dr. H. A. Johnson had been in the habit, for the past eight years, of using the potassium bromide in cases where he did not desire to give opium, to allay irritability, and in the early stage of diarrhœa. He knew of similar practice in the U. S. Army. The irritability is not a primary disease, but is the result of less tonicity. I am quite sure the bromide will check the disease; it stimulates the urinary excretion, allays the nervous system, the irritation and peristaltic action of the bowel. The potassium bromide may be given, to a child one year of age, in two to four-grain doses, every four hours, and in pretty full doses as compared with adults; often enough to procure rest.

Dr. Etheridge had had limited experience with the bromide in the treatment of diarrhœa in children. He had found that the number of the discharges in the first twenty-four hours had not lessened, but their character had improved, and the child was better. If the use of the bromide was continued after the child was better, you would get brominism, with its eruption.

Upon motion, the Society adjourned.

*Transactions at Regular Meeting, Aug.* 10, 1874.

The Society met as usual in Parlor No. 1 of the Grand Pacific Hotel.

Dr. A. Fisher was, upon motion, called to preside, the two Presidents being unavoidably absent when the hour for business arrived.

The names of Drs. H. N. Hurlbut and F. L. Wadsworth were proposed for membership, and referred to the Board of Censors.

Dr. Etheridge read an exceedingly interesting article, which he had translated from the *Progres Medical*, being a paper by Dr. H. Chauppe on "The Therapeutical Study of Ipecac, when used in the Sweats of Pulmonary Phthisis." The following is a brief summary:

The history and details of twelve cases of phthisis, in its various stages, were given with much minuteness, and were divided into groups illustrating the results, more or less favorable in degree and duration of relief. Each group of cases was reviewed, from which certain deductions were drawn, and objections noted and refuted. To one objection, that the injections of ipecac act, in sweatings, only by means of the tannic acid which they hold in solution, it was answered that in two cases where tannin had been given in rather large doses, no amelioration was produced; the ipecac clysters promptly arrested the sweats. The atropia sulphate, drachm doses of quinia tannate, or six-grain doses of tannin itself, had not controlled these cases of sweating. In these twelve cases, ipecac was found to act quite constantly in night sweats, with much benefit. It should be used in rebellious cases, when the excellent remedies above mentioned prove futile.

The different types and forms of sweating were then adverted to, and attention called to a point long neglected: Are the sweats preceded, or not, by fever? *Does the exhaustion follow the cold or hot sweats?* Probably in these two cases of sweating, the treatment for the sweats ought not to be the same. This problem still awaits solution. In most of the cases where ipecac has a good effect, the fever is noticed to precede the sweats.

*Mode of Administering—Doses.* We believe that the exhibition of ipecac, per rectum, is a veritable progress in treatment, and this method is due to M. Bourdon. It may be given in large doses, and for a long time, without producing a single mishap. We have used this form of decoction of ipecac: R. Powdered ipecac root, grains 154; pure water, grammes 100, by weight. Boil until reduced to 30 grammes. Filter this decoction. Prepare a second decoction, analogous to the first one, mix the two, and add five to ten drops of Sydenham's laudanum. It is better to use a glass syringe. Give one injection only, and as late as possible at night. Where the Brazilian potion was used, it consisted of the decoction of ipecac prepared as above, but reduced to 45 grammes, to which was added of the syrup of ether 15 grammes. Divide into three parts, and give one part every quarter of an hour.

CONCLUSIONS.

1. Ipecac, even in large doses, administered per rectum, does not produce vomiting or gastric disturbance.

2. In many cases of infantile diarrhœa, injections of ipecac act favorably.

3. Diarrhœa of the tuberculous was beneficially influenced; often for a long time cured, by ipecac in large doses. The autopsies afterwards made in some of these cases, displayed no organic lesions of the mucous membrane of the intestines.

4. The action of ipecac, upon phthisical sweats, has been the most frequently favorable.

5. Ipecac seems to act by absorption.

6. The tannin is in too small a quantity (gr. 1½) in the decoction, to have the therapeutical effect attributed to it.

Dr. D. A. K. Steele, of the Cook County Hospital, reported four cases of land scurvy. One patient was a Norwegian, a cabinet-maker, who had been living in abject poverty for several years. He was very melancholic, and neglected personal cleanliness. The disease had been progressing for many months. His bowels were very costive; had moved twice in four weeks. His condition was one of extreme anæmia and debility. Gums swollen, mastication difficult, limbs œdematous and ecchymotic; at the knees, the swelling prevented flexion and extension. With the first sound of the heart, there was a soft murmur.

The other three cases were in boys eight and nine years of age, who had been living in an asylum, whose wards were greatly overcrowded, and whose dietary had been very deficient in vegetables. Symptoms and condition very similar to the first case, but less severe; though one of the boys had lost two teeth. The treatment consisted in a liberal allowance of vegetables; also acid drinks; also ten drops of tr. ferri muriatis every four hours, to the boys; twenty drops to the adult; also a gargle of potassium permanganate, one grain to the fluid ounce. Dr. Steele said that scurvy is a very difficult disease to cure, but thta these patients, particularly the boys, were fully convalescent.

Dr. Hyde brought before the Society the subject of prescribing proprietary medicines or remedies—a practice so largely on the increase in this city that six out of nine of the prescriptions received by the average grade of drug-stores (perhaps not so large a proportion by the best class of stores) ordered proprietary medicines, and even named the makers of the goods. He entered his protest against such practice, and against the swarm of traveling salesmen who throng into the offices, peddling their samples of medicines, repeating their glib stories with tiresome sameness; thus they go the rounds, month after month, and leave their bottles behind them, hoping by donating a few samples to increase the demand for their goods. What is the effect upon the wholesale drug-dealer? His stock will be made up of *proprietary medicines*, one-sixth; *patent medicines*, one-third; *paints* and *oils*, one-third; and the balance, one-sixth, of *drugs* and *medicines for prescriptions.*

Mr. A. E. Ebert, a leading druggist of this city, editor of *The Pharmacist*, and lately President of the American Pharmaceutical Association, was present, and, by request, addressed the Society substantially as follows: Twenty years ago, the wholesale druggists sold—in equal quantities—drugs and patent medicines. Fifteen years ago, the fluid extracts and preparations of the so-called "elegant pharmacy," became the fashion. In 1860, the Pharmacopœia fixed the strength of fluid-extracts, but no honest, conscientious pharmacist can prepare them, and compete successfully with the dishonest manufacturer. Mr. Ebert exposed the vagaries of the elixir and the sugar-coated pill business, and various adulterations; one of these, consisted in taking the silver coin of the country and making the same into nitrate of silver; in one instance, he found one ounce of pure copper in thirteen of nitrate of silver. The makers of many articles of "elegant pharmacy" cannot by any exertion sell their goods in their own cities. The people are learning to do without physicians and can go to the stores, look at the medicines, and buy whatever they choose. It injures the physician, and degrades the pharmacist; makes middlemen of them, and kills off all incentive to accurate, scientific, honorable pharmacy. The only way in which to quell this evil, is to cease mentioning names of makers, and to order only the officinal remedies authorized by the U. S. Pharmacopœia.

Dr. Hay said that the remarks of Dr. Hyde and Mr. Ebert suggested the relations which ought to exist between the druggist, pharmacist and physician. After referring to the attacks, lately made by the secular papers, upon the profession, alleging that physicians were defrauding the public and their patients by collusion with druggists from whom they received a commission on prescriptions, and other perquisites, he said that if these charges are true, it is quite time that we should relieve ourselves of the odium; if false, let us refute them. He was interested in the profession, its standing, its integrity—honor—and the confidence it receives from the public, which is the sole basis on which it rests. He had made some inquiries and investigations, which had induced him to think that the charges, as made, were true. He would, therefore, introduce the following preamble and resolutions, which, upon motion, were passed without one negative vote, in a meeting of above the average attendance of members:

WHEREAS, The medical profession of Chicago, on several recent occasions, has been charged in the *Chicago Times* with collusion with pharmacists for the purpose of extorting money from their patients; and,

WHEREAS, This collusion is alleged to be effected in various ways: such as—

1. The use of prescription papers bearing the business cards of pharmacists;
2. The occupation of offices free of, or at nominal rent, adjacent to or belonging to pharmacists;
3. The writing of private formula understood by certain pharmacists exclusively;
4. The acceptance of commissions upon prescriptions from pharmacists; and,

WHEREAS, The practices above designated, although deprecated by many always, have been maintained by many others innocently and in good faith, unsuspicious of their abusive application; therefore, be it

*Resolved*, That the Society of Physicians and Surgeons of the City of Chicago recognize the fact that the practices above designated tend to the degradation and demoralization of the medical profession, and to the diminution and withdrawal of public confidence upon which its existence depends.

*Resolved*, That the members of the Society pledge themselves, as individuals and as an organization, to discontinue the practices above designated, so far as they may have adopted them, and to discountenance them in others so far as their influence may extend.

*Resolved*, That the Society regards the acceptance of commissions upon prescriptions, by physicians, from pharmacists, as positively disreputable and dishonest, and to be deemed a sufficient cause for the rejection of an applicant for, or for the expulsion of a holder of membership herein.

Dr. Jackson, in seconding the adoption of the resolutions said that it was a growing evil, and that so far as it rests with us, we should shake it off.

Dr. Hamill—I heartily concur with the sentiment of the resolutions.

Dr. Etheridge—The evil is imaginary rather than a real one. He had called on a few of the leading druggists, and found that they never allowed commissions. He was astonished to find so little of it. It is the charlatans who bring these charges upon us. Are we not fighting a phantom?

Following remarks by Doctors Hamill and A. Fisher, Dr. Jackson said, that he hoped the practice was much less than was alleged, but thought that where there was so much smoke there must be some fire; and alluded to an incident where a medical friend of his had had a bottle of perfumery and a roll of bank bills presented to him by an apothecary, as a commission for prescriptions. The money was indignantly returned, and his friend forever afterwards withheld his patronage from that druggist.

The following resolution was proposed by Dr. Hyde and seconded by Dr. Hay:

*Resolved*, That a committee of three be appointed by the chair to co-operate with a similar committee from the Chicago College of Pharmacy (should such be appointed) in order to consider what, if any, measures are requisite in order to correct the great and growing evil in our midst of the prescribing of proprietary medicines, by name or otherwise, and of the accepting of samples of such medicines as are distributed in this city by the agents of eastern wholesale drug houses. And that such joint committees (should concurrence be had) report in full to the Society for such action in the premises as may seem to be desirable.

Mr. Ebert was of the opinion that the College of Pharmacy would co-operate.

The resolution was passed by an unanimous vote, and Doctors Hyde, Hay and Etheridge were appointed as such committee.

Dr. Jackson, on behalf of the Fee-Bill Committee, reported a schedule of rates for fees. The Committee were directed to confer with a similar one of the Chicago Medical Society.

Upon motion, the Society adjourned.

**Chicago Medical Society.** *Transactions at the Meeting of July* 20, 1874. Reported by WILL T. MONTGOMERY, M.D.

The Society met as usual in the parlor of the Gault House, the President, Dr. Quine, in the chair.

In the absence of the Secretary, Dr. Graham was elected Secretary *pro tem.*

The special order for the evening being report of Section on Obstetrics, Dr. A. B. Strong read a paper on Fœtal Pulsation. Dr. D. A. K. Steele read a supplemental report on the same subject. It was moved and carried that both papers be published in the report of the Society.

In the discussion of the papers, Dr. C. M. Fitch said he had made examinations in eleven cases for the purpose of determining sex, and predicted correctly in nine. When the pulsations were over 140 per minute, he predicted a girl; when under 130, a boy. Between these two numbers, he was doubtful. He thought a stethoscope, with a metallic bell, preferable in these examinations.

Dr. Paoli could not see any practical importance in determining the sex of the fœtus in utero. He was not aware that the sex made any difference with regard to preparations of clothing, etc. He could see how it might be of importance to crowned heads to know the sex before birth, in that they could then be prepared to celebrate the birth of prince or princess, as the case might be.

Dr. Bridge said while the determining of sex might not be of practical importance, the facts which may be gained in reference to the health and presentation of the child, and condition of the placenta, are of much importance.

Dr. T. D. Fitch—We may very well say that these investigations are of no practical interest, but they are of much scientific interest. He did not think it practically important to determine before birth the condition of the child or placenta, as the physician should be prepared for any emergency that might arise.

Dr. Seely—It may not be necessary to determine the sex before birth, but if we are able to do this we may gain the confidence of our patients, which is not to be despised.

Dr. Graham agreed with Dr. Fitch as to the scientific interest of these investigations.

Dr. Montgomery had made examinations in a number of cases with reference to determining sex, and was generally correct in predicting a girl when the pulsations were over 130, and a boy, when they were under 130.

Dr. Strong said that in the first eight cases which he examined he was correct in predicting the sex, and he thought a greater number of cases were required if we wished to aim at conclusions of value. He referred to a case of contracted pelvis, reported by Dr. Wilson, in which the determining of sex in utero was of practical importance.

Dr. Steele thought the determining of sex in utero might be of value in a medico-legal point of view.

Dr. Van Buren wished to know if there is any truth in the theory that gestations of mothers carrying male children are longer than of those carrying female. He also wished to know what effect protracted labors have upon the fœtal pulsations.

Dr. C. M. Fitch had seen a child delivered after a protracted labor of forty hours. The child proved to be idiotic, and he thought this was the result of the long-continued pressure.

Dr. Stilliaus had seen a similar case, and thought the injurious effect of continued pressure was an argument against the use of ergot.

Dr. T. D. Fitch said in reference to the length of gestations he had observed that those of mothers carrying male children were longest, and if the mother went over her time, he was generally correct in predicting a boy.

President had not heard of any reliance being placed on the length of time of gestation. He had made a number of observations in reference to determining sex by auscultation, and had usually been correct. The position could not be so reliably determined by this as by external manipulation. He thought inflammation of the placenta could be diagnosed by repeated auscultations.

Dr. Bartlett—An exceedingly slow pulse, as seventy to eighty, is very unfavorable to the child, and when it is observed, delivery should be effected as soon as possible. He had been called, within the last week, a distance of four miles, to see a lady, and when he arrived at her house learned that all she wanted him for was to tell her whether she was going to have a boy or girl.

Dr. Foster does not believe the length of time of gestation is a true indication of sex. He recently attended a lady on the 285th day, and delivered her of a girl.

Dr. Bridge gave an explanation of the marked influence the contractions of the uterus exercise over the fœtal pulsations. He thought the great diminution in the number of pulsations at the height of contraction was due to the circulation being cut off so that the blood in the placenta and fœtus became poisoned from non-aeration.

After miscellaneous business was disposed of, the Society adjourned.

*Transactions at Meeting of Aug.* 3, 1874.

Society met as usual, in the parlors of the Gault House, the President, Dr. Quine, in the Chair.

Dr. Quine read a paper presenting the most important facts from a record of a number of cases of puerperal metritis, metro-peritonitis, and puerperal septicæmia, which had occurred in Cook County Hospital. He said he wished to present the paper as introductory to the subject of puerperal fever which he hoped to consider more fully at a subsequent meeting. In the cases presented, the line of distinction between the three diseases was pretty clearly drawn. With reference to treatment, blisters and poultices to the abdomen were used in all cases. The chief treatment in the cases of simple metritis was veratrum, or aconite combined with opium sufficient to relieve pain, and attention to cleanliness. The cases of puerperal septicæmia were treated substantially as typhoid fever. The opium treatment was used in the cases of metro-peritonitis. In two cases that recovered, dr. ss. of morphine was given for several days in succession. The pulse was kept between four and eight in the minute. To one patient gr. vij of morph. were given several successive hours, and for two days previously gr. v were administered every hour. The morphine was gradually withdrawn three days before death, and while it was used, no effect was noticed on the pulse or respiration. Veratrum was faithfully tried, but invariably increased the frequency and diminished the fullness of the pulse Quinine had a marked effect in diminishing the intensity of the fever. His experience is decidedly in favor of opium and quinine.

Dr. C. M. Fitch had seen five well marked cases of puerperal fever, and all were fatal. He related a case following an abortion, which forcibly impressed him with the contagiousness of the disease. All he had read on the subject had been eminently unsatisfactory.

Dr. Jacobson had seen local depletion by means of leeches to the abdomen used a great deal, but has not been encouraged to use it in his own practice. He has seen good effect from the use of collodion to the abdomen; said there is a great difference in regard to prognosis in different epidemics. Little can be done after the disease is well established. We cannot exercise too much care in regard to prevention by contagion. He thought the disease often arose from products of the uterus retained by inertia, and recommended the use of ergot in cases of faulty contractions. Referred to two cases in which decayed fœtuses seemed to be the cause of the disease.

Dr. T. D. Fitch had had but little experience with the disease in private practice. He agreed with Dr. Jacobson in reference to the importance to prophylaxis. Patients may be infected by the physician after making autopsies or attending erysipelatous cases. The greatest care should be observed in disinfecting the hands and clothing. He coincided with the views of Dr. Jacobson with regard to the retained products of the uterus and ergot. He is particular to give ergot in all cases in which there has previously been post partum hemorrhage or inertia. Injury to the parts may be an exciting cause of the disease. As regards mortality, much depends upon the severity of the epidemic. In the treatment of puerperal cases he secures an early evacuation of the bowels, and prefers castor oil. Does not use vaginal injections unless the discharge are offensive. He usually begins the treatment of the fever by giving veratrum in gtts iv doses, and increases the dose until the pulse is brought down to 60 or 70. In malignant cases veratrum will not reduce the pulse, and stimulants are indicated. He uses blisters but never bleeds; withdraws the veratrum as the case progresses. Does not believe in heroic doses of opium, but gives sufficient to control pain.

Dr. Stilliaus had never had a case of fever in a patient which he had delivered, and had flattered himself that it was due to his management of his patients. He uses vaginal injections in all his labor cases.

Dr. Strong had seen eight cases, six died and two recovered. One of the cases that recovered was leeched; and in the other, stimulants and quinine were used.

Dr. Paoli said that we labor under the delusion that these cases sometimes recover. He did not believe any well marked cases ever recover, and he did not believe any gentleman would swear that he had seen such recover. He did not believe that it was always possible to distinguish between the different puerperal diseases. Condemns bleeding, and says that veratrum does not have any curative effect, but only controls the severity of the symptoms. So with quinine. Did not think any medicine had any curative effect in this disease. A thorough examination will always discover pus in the blood of true puerperal fever cases.

Dr. Millard had never been able to distinguish between puerperal fever and peritonitis. Had had success from venesection in the treatment of these cases. Thinks the thorough contraction of the uterus after delivery important.

Dr. Taggart had seen the veratrum and opium treatment used in an epidemic in Buffalo with unsatisfactory results.

Dr. Van Buren does not know what puerperal fever is. He had been taught from books that it is an inflammation. If we knew just what it is, we might treat it intelligently. He said if there is any truth in the poison theory, he thought it was important to secure firm contraction of the uterus. Had never seen but one case of the fever, and that died.

Dr. Etheridge inquired as to the large doses administered to the case of Dr. Quine, if the patient really took gr. vij of morphia for a number of hours in succession.

Dr. Quine said the dose was gradually increased up to gr. vij, and repeated a number of times. Such heroic doses were only administered to the one case. He said some important points had been brought out in the discussion that were not alluded to in the paper. First, as to the contagiousness and nature of the disease. He had used the term generically. Does not think there is any connection between puerperal fever and other inflammations. The poison may enter the system through any open blood vessels.

He thought puerperal fever might occur before confinement, and referred to a case in point. Had never communicated the disease, and latterly had not used great care. The specific virus may be readily communicated by one and not by another. One midwife had recently furnished him eleven malignant cases. Hê had seen cases in which the injudicious use of cathartics seemed to aggravate the disease and hasten a fatal termination. He had used quinine and nux vomica in cases of inertia with good effect. Quinine is the most efficient remedy in restraining the disease, and he administers to cinchonism. Had used stimulants in most cases. Pain is not a marked symptom, and he did not give opium for its anodyne effect but for the specific effect he thought it had upon the inflammation. He was as sure as he could be, without an autopsy, that he had seen true cases of puerperal fever recover.

Dr. Mary Thompson asked, Is it right for a physician to go from a case of eruptive fever to a case of confinement?

Dr. Paoli answered, in his usual positive manner, *No.*

After miscellaneous business was disposed of, a motion to adjourn prevailed.

